# SIRT5 mitigates metabolic abnormality in murine models of metabolic dysfunction-associated steatotic liver disease

**DOI:** 10.1093/lifemedi/lnag001

**Published:** 2026-01-20

**Authors:** Min Xiao, Juncheng Zhao, Zixuan Dou, Xiangyu Chen, Sunyuntao Xu, Yu Zhang, Hongxuan Fan, Xudong Chen, Ping Zhang, Zhen Huang, Boda Zhou, Taotao Wei

**Affiliations:** State Key Laboratory of Biomacromolecules, Institute of Biophysics, Chinese Academy of Sciences, Beijing 100101, China; State Key Laboratory of Biomacromolecules, Institute of Biophysics, Chinese Academy of Sciences, Beijing 100101, China; College of Life Sciences, University of Chinese Academy of Sciences, Beijing 100049, China; Department of Rare Diseases and State Key Laboratory of Complex Severe and Rare Diseases, Peking Union Medical College Hospital, Peking Union Medical College & Chinese Academy of Medical Science, Beijing 100730, China; State Key Laboratory of Biomacromolecules, Institute of Biophysics, Chinese Academy of Sciences, Beijing 100101, China; College of Life Sciences, University of Chinese Academy of Sciences, Beijing 100049, China; State Key Laboratory of Biomacromolecules, Institute of Biophysics, Chinese Academy of Sciences, Beijing 100101, China; State Key Laboratory of Biomacromolecules, Institute of Biophysics, Chinese Academy of Sciences, Beijing 100101, China; College of Life Sciences, University of Chinese Academy of Sciences, Beijing 100049, China; Department of Cardiology, Beijing Tsinghua Changgung Hospital, School of Clinical Medicine, Tsinghua University, Beijing 102218, China; Ministry of Education Key Laboratory of Protein Science, Beijing Advanced Innovation Center for Structural Biology and Frontier Research Center for Biological Structure, Tsinghua-Peking Joint Center for Life Sciences, School of Life Sciences, Tsinghua University, Beijing 100084, China; Department of Cardiology, Beijing Tsinghua Changgung Hospital, School of Clinical Medicine, Tsinghua University, Beijing 102218, China; Department of Hepatobiliary Surgery, National Cancer Center/National Clinical Research Center for Cancer/Cancer Hospital, Chinese Academy of Medical Sciences & Peking Union Medical College, Beijing 100021, China; Department of Cardiology, Beijing Tsinghua Changgung Hospital, School of Clinical Medicine, Tsinghua University, Beijing 102218, China; State Key Laboratory of Biomacromolecules, Institute of Biophysics, Chinese Academy of Sciences, Beijing 100101, China; College of Life Sciences, University of Chinese Academy of Sciences, Beijing 100049, China

Dear Editor,

Metabolic dysfunction-associated steatotic liver disease (MASLD) can progress to metabolic dysfunction-associated steatohepatitis (MASH), hepatic fibrosis, and, ultimately, cirrhosis and/or hepatocellular carcinoma [1]. During the early stage of MASLD, fatty acid β-oxidation and tricarboxylic acid cycle activity are elevated in the mitochondria of hepatocytes, providing energy and substrates to support *de novo* lipogenesis. However, with the progression to MASH, lipotoxicity-induced mitochondrial damage and dysfunction lead to an imbalance between lipid accumulation and disposal, and contribute to inflammation and fibrosis [2]. Impaired metabolism causes the accumulation of acetyl-CoA, malonyl-CoA, and succinyl-CoA, which act as donors for protein acylation. We observed elevated lysine malonylation and succinylation in type 2 diabetes mellitus (T2DM) [3]; however, functional studies of lysine acylation in metabolic diseases have been lagging behind.

This study aimed to explore the potential role of protein acylation in MASLD. We began by analyzing lysine malonylation and succinylation in liver tissues of mice fed with a high-fat diet (HFD), a well-established murine dietary model for MASLD [4]. Using a tandem mass spectrometry workflow that involves the enrichment of malonylated and succinylated peptides (Fig. 1A), we identified and quantified 1123 unique malonylated peptides corresponding to 548 proteins and 3420 unique succinylated peptides mapping to 934 proteins. Compared with the normal diet (ND) group, the HFD group exhibited a significant increase in the levels of 72 malonylated and 271 succinylated peptides (≥2-fold change, *P *< 0.05). Gene ontology (GO) analysis revealed that proteins showing elevated malonylation and succinylation were predominantly involved in metabolic pathways (Fig. 1B and 1C).

**Figure 1.**
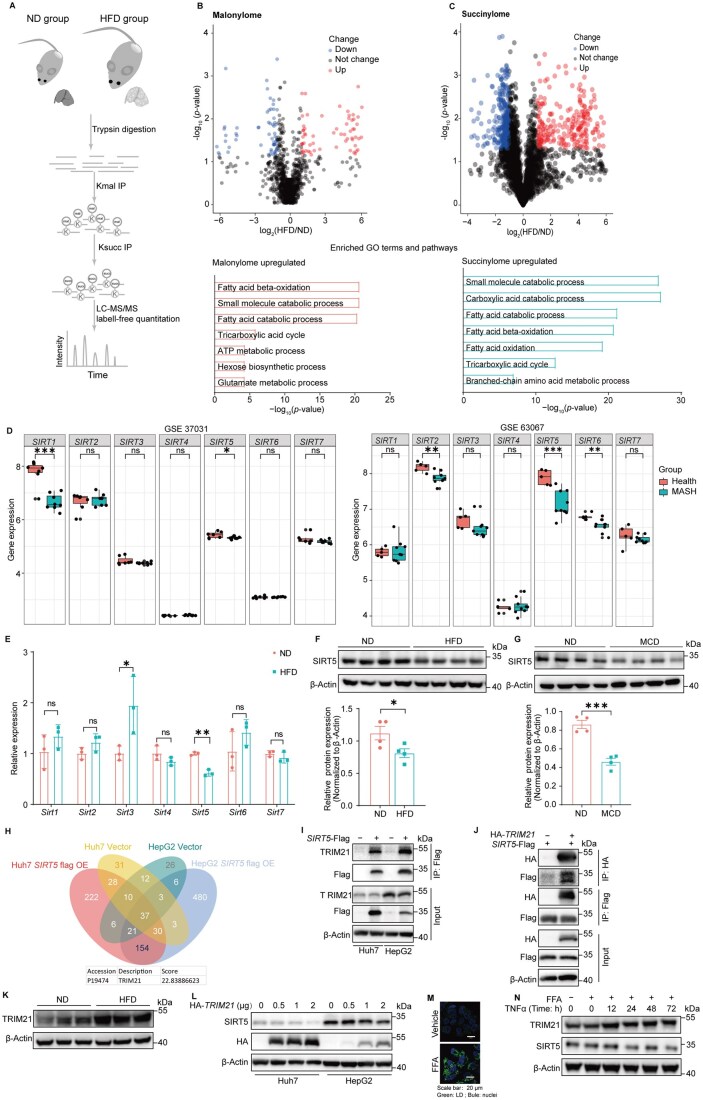
**SIRT5 is down-regulated in MASLD**. (A) Schematic of the experimental workflow for proteomic identification of the hepatic malonylome and succinylome in C57BL/6J mice after 14 weeks on ND or HFD (*n* = 3). (B) Fold change distribution of Kmal peptides between ND- and HFD-fed C57BL/6J mice after 14 weeks. Kmal peptides with the threshold of ratio HFD/ND ≥ 2 and HFD/ND ≤ 0.5 (*P* < 0.05) are highlighted; representative GO terms and pathways enriched in proteins exhibiting upregulated lysine malonylation in HFD-fed mice are shown. (C) Fold change distribution of Ksu peptides between ND- and HFD-fed C57BL/6J mice after 14 weeks. Ksu peptides with the threshold of ratio HFD/ND ≥ 2 and HFD/ND ≤ 0.5 (*P* < 0.05) are highlighted; representative GO terms and pathways enriched in proteins exhibiting upregulated lysine succinylation in HFD-fed mice are shown. (D) *Sirtuin* mRNA expression profiles were obtained from the NCBI Gene Expression Omnibus (GEO) datasets GSE37031 and GSE63067. Between-group differences were assessed using the Wilcoxon rank-sum test. *P < 0.05, **P < 0.01, ***P < 0.001 compared with the healthy group. (E) qPCR analysis of hepatic *Sirtuin* mRNA in C57BL/6J mice after 14 weeks on ND or HFD (*n* = 3). *P < 0.05, **P < 0.01 compared to ND. (F) Western blot analysis and quantification of hepatic SIRT5 in C57BL/6J mice after 14 weeks on ND or HFD (*n* = 4). *P < 0.05 compared to ND. (G) Western blot analysis and quantification of hepatic SIRT5 in C57BL/6J mice after 14 weeks on ND or MCD (*n* = 4). ***P < 0.001 compared to ND. (H) Venn diagram presenting the potential unique overlapping SIRT5-interacting proteins identified through LC–MS/MS analysis. Empty Vector cells served as the negative control. (I) Huh7 and HepG2 cells stably expressing *SIRT5*-Flag were immunoprecipitated with an anti-Flag antibody. (J) Lysates from HEK293T cells transfected with HA-*TRIM21*, *SIRT5*-Flag, or vector control (−) as indicated were immunoprecipitated using anti-Flag antibody and anti-HA antibody. (K) Western blot analysis of hepatic TRIM21 protein in C57BL/6J mice after 14 weeks on ND or HFD (*n* = 3). (L) Western blot analysis of whole-cell lysates from Huh7 and HepG2 cells transfected with increasing amounts of HA-*TRIM21* constructs. (M) Representative confocal microscopy images of Huh7 cells stained with a lipid droplet probe, both under native conditions and in response to stimulation with an FFA mixture (200 µmol/L palmitic acid [PA] and 400 µmol/L oleic acid [OA]). (N) Western blot analysis of whole-cell lysates from HepG2 cells treated with a FFA mixture (200 µmol/L PA and 400 µmol/L OA) and TNF-α (50 ng/mL) for the indicated durations.

Since sirtuins orchestrate the elimination of protein acylation, we analyzed their expression profiles in patients diagnosed with MASH. Results shown in Fig. 1D revealed a notable decline in the expression of *SIRT5* among MASH patients, and its expression was negatively correlated with the severity of steatohepatitis (Fig. S1A). Single-cell RNA sequencing (scRNA-seq) of livers from mice fed with a Western diet revealed that *Sirt5* was specifically downregulated in hepatocytes (Fig. S1B). Consistently, both the *Sirt5* mRNA (Fig. 1E) and the SIRT5 protein (Figs.1F and S1C) levels were decreased in the liver of HFD mice, but not in other organs (Fig. S1D). In mice fed with methionine/choline-deficient diet (MCD), hepatic SIRT5 was also reduced (Fig. 1G).

To elucidate the mechanisms that control SIRT5 expression in MASLD, we profiled the protein–protein interaction partners of SIRT5. Huh7 and HepG2 cells were transfected with Flag-tagged *SIRT5*, followed by immunoprecipitation and LC–MS/MS shotgun proteomics. The E3 ubiquitin ligase tripartite motif-containing protein 21 (TRIM21) was identified as the top interactor of SIRT5 (Fig. 1H). Co-IP validated the interaction between SIRT5 and TRIM21 (Figs. 1I and 1J, and S1E). Given that TRIM21 mediates the degradation of SIRT5 [5], we hypothesized that it might control the down-regulation of SIRT5 in MASLD. *TRIM21* expression was significantly elevated in liver specimens from MASLD patients (Fig. S1F). Consistently, TRIM21 levels were profoundly increased in the livers of HFD mice compared to ND controls (Fig. 1K). Overexpression of TRIM21 decreased SIRT5 levels in both Huh7 and HepG2 cells (Fig. 1L). To explore the molecular mechanisms underlying TRIM21 induction in MASLD, we treated cells with TNF-α and free fatty acids (FFA), factors that have been linked to MASLD progression. FFA efficiently induced lipid accumulation in hepatic cells (Fig. 1M) and, in combination with TNF-α, synergistically upregulated TRIM21 expression while further suppressing SIRT5 protein levels (Figs. 1N and S1G).

SIRT5 exhibits robust demalonylase and desuccinylase activities. Given the observed down-regulation of SIRT5 expression levels and elevated lysine malonylation and succinylation in MASLD, we hypothesized that the restoration of SIRT5 expression would mitigate the dysregulated lipid and glucose metabolism in MASLD. To test this hypothesis, we developed mice with liver-specific overexpression of SIRT5 (referred to as Liver *Sirt5* OE mice) (Fig. 2A), and induced MASLD with HFD. Since patients with MASLD have a higher prevalence of T2DM, we performed glucose tolerance tests and insulin tolerance tests (ITT). In HFD-fed Liver *Sirt5* OE mice, basal blood glucose levels were reduced compared with HFD-fed wild-type (WT) mice, and glucose tolerance was improved (Fig. 2B). ITT experiments indicated that Liver *Sirt5* OE mice exhibited enhanced insulin sensitivity (Fig. 2C). In terms of lipid metabolism, Liver *Sirt5* OE mice fed with HFD showed significantly lower levels of plasma triglycerides (TG), total cholesterol (TC) and low-density lipoprotein (LDL), compared with WT mice fed with HFD (Fig. 2D–F). Histological analysis confirmed reduced hepatic steatosis in Liver *Sirt5* OE mice (Fig. 2G and 2H). Consistent with this histological improvement, the HFD‑induced increase in liver weight was significantly attenuated in Liver *Sirt5* OE mice compared with WT mice (Fig. 2I). The activities of alanine aminotransferase (ALT) and aspartate aminotransferase (AST) were also decreased in Liver *Sirt5* OE mice (Fig. 2J and 2K). The ultrastructure of mitochondria from Liver *Sirt5* OE mice remained clear, with reduced lipid droplet accumulation (Fig. 2L). Concomitantly, we found reduced liver TG in Liver *Sirt5* OE mice in response to HFD (Fig. 2M). *In vitro* experiments also demonstrated that SIRT5 prevents excessive lipid deposition caused by FFA stimulation in primary hepatocytes (Fig. 2N).

**Figure 2.**
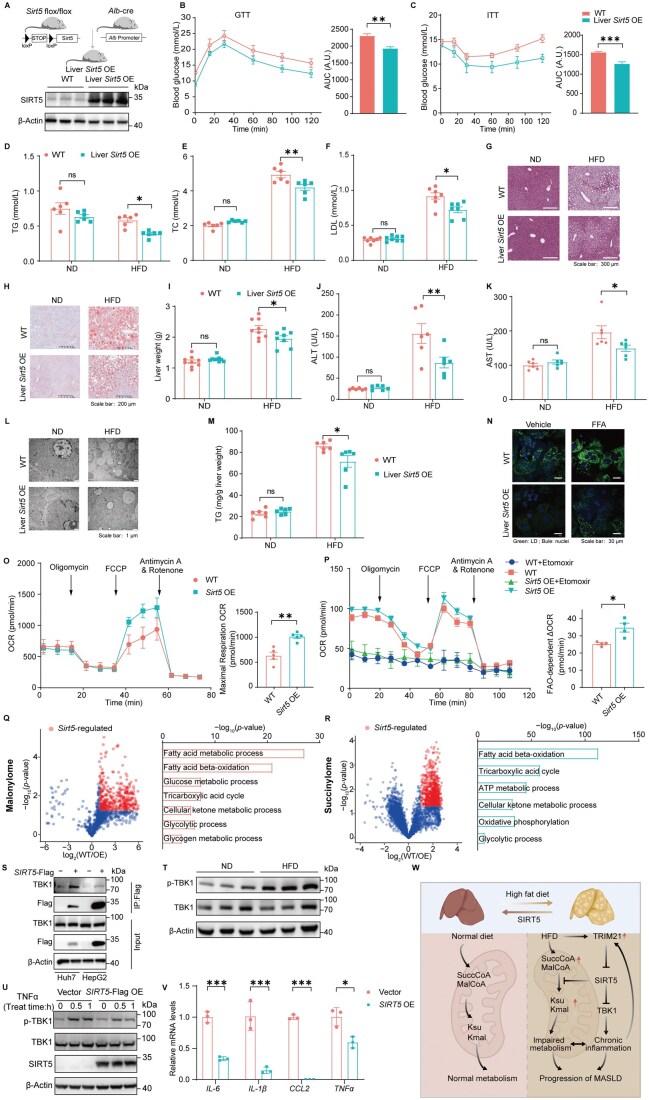
**Overexpression of SIRT5 demalonylates and desuccinylates proteins to improve glucose and lipid metabolism**.(A) Schematic diagram of the breeding strategy to generate Liver *Sirt5* OE mice. (B) GTT and quantification of AUC in WT and Liver *Sirt5* OE mice after 14 weeks on HFD (*n* = 5). **P < 0.01 compared to WT. (C) ITT and quantification of WT and Liver *Sirt5* OE mice after 14 weeks on HFD (*n* = 5). ***P < 0.001 compared to WT. (D) Serum TG levels of WT and Liver *Sirt5* OE mice after 14 weeks on ND or HFD (*n* = 6). *P < 0.05 compared to WT. (E) Serum TC levels of WT and Liver *Sirt5* OE mice after 14 weeks on ND or HFD (*n* = 6). **P < 0.01 compared to WT. (F) Serum LDL levels of WT and Liver *Sirt5* OE mice after 14 weeks on ND or HFD (*n* = 7). *P < 0.05 compared to WT. (G) Representative images of HE staining of liver tissue from WT and Liver *Sirt5* OE mice after 14 weeks on ND or HFD. (H) Representative images of Oil Red O staining of liver tissue from WT and Liver *Sirt5* OE mice after 14 weeks on ND or HFD. (I) Liver weight of WT and Liver *Sirt5* OE mice after 14 weeks on ND or HFD (*n* = 8). *P < 0.05 compared to WT. (J) Serum ALT levels of WT and Liver *Sirt5* OE mice after 14 weeks on ND or HFD (*n* = 6). **P < 0.01 compared to WT. (K) Serum AST levels of WT and Liver *Sirt5* OE mice after 14 weeks on ND or HFD (*n* = 6). *P < 0.05 compared to WT. (L) Representative electron microscopy images of hepatocytes from WT and Liver *Sirt5* OE mice after 14 weeks on ND or HFD. (M) Comparison of hepatic triglyceride (TG) levels in WT and Liver *Sirt5* OE mice after 14 weeks on ND or HFD (*n* = 8). *P < 0.05 compared to WT. (N) Representative confocal microscopy images of primary hepatocytes stained with a lipid droplet probe, both under native conditions and in response to stimulation with FFA mixture (200 µmol/L PA and 400 µmol/L OA). (O) Comparison of OCR in primary hepatocytes from WT and Liver *Sirt5* OE mice in response to stimulation with FFA mixture (200 µmol/L PA and 400 µmol/L OA) (*n* = 5). **P < 0.01 compared to WT. (P) Quantification of the change in OCR (ΔOCR; a proxy for fatty acid oxidation [FAO]) in hepatocytes (*n* = 4). *P < 0.05 compared to control cells. (Q) Volcano plot of Kmal peptides; red dots indicate *Sirt5*-regulated sites, defined by a fold change (WT/OE) ≥ 2 and *P* < 0.05; GO and pathway enrichment analysis of proteins with *Sirt5*-regulated lysine malonylation sites in HFD-fed mice. (R) Volcano plot of Ksu peptides; red dots indicate *Sirt5*-regulated sites, defined by a fold change (WT/OE) ≥ 2 and *P* < 0.05; GO and pathway enrichment analysis of proteins with *Sirt5*-regulated lysine succinylation sites in HFD-fed mice. (S) Huh7 and HepG2 cells stably expressing *SIRT5*-Flag were immunoprecipitated with an anti-Flag antibody. (T) Western blot analysis of hepatic phosphorylated TBK1 (p-TBK1) in C57BL/6J mice after 14 weeks on ND or HFD (*n* = 3). (U) Western blot analysis of phosphorylated TBK1 (p-TBK1) in HepG2 cells overexpressing *SIRT5* (*SIRT5* OE) and control cells (Vector) treated with TNF-α (50 ng/mL) for the indicated times. (V) qPCR analysis of inflammatory cytokine mRNA expression in *SIRT5* OE and control cells (Vector) treated with FFA mixture (200 µmol/L PA and 400 µmol/L OA) and TNF-α (50 ng/mL) for 24 h (*n* = 3). ***P < 0.001, *P < 0.05 compared to Vector. (W) Schematic of SIRT5 mitigating metabolic abnormalities in murine models of MASLD.

To examine whether mitochondrial respiration was affected by SIRT5, we measured the oxygen consumption rate (OCR) of primary hepatocytes from Liver *Sirt5* OE and WT mice, using glucose as substrate. As indicated in Fig. 2O, SIRT5 elevated the maximal OCR capacity, suggesting that it enhances mitochondrial respiratory function. SIRT5 also increased the capacity for fatty acid β-oxidation (Fig. 2P), suggesting that SIRT5 promotes glucose and lipid metabolism by boosting mitochondrial bioenergetic capacity.

We conducted proteomic analysis to compare the levels of malonylated and succinylated peptides in Liver *Sirt5* OE and WT mice fed either ND or HFD. Malonylation increased in 404 out of 1137 peptides (WT/Liver *Sirt5* OE ratio ≥ 2, *P *< 0.05), defining *Sirt5*-regulated demalonylation targets. Succinylation increased in 772 out of 3872 peptides under the same thresholds, defining *Sirt5*-regulated desuccinylation targets. GO enrichment showed these proteins are enriched in glucose and lipid metabolism, indicating that Liver *Sirt5* OE drives demalonylation and desuccinylation of metabolic proteins (Fig. 2Q and 2R). Notably, we found that 35 malonylated peptides in WT hepatocytes due to HFD were demalonylated in SIRT5 high-expressing hepatocytes, and 52 peptides showed a similar trend for succinylation. Pathway enrichment analysis showed these overlapped proteins were mainly enriched in metabolism pathways, such as fatty acid β-oxidation, and tricarboxylic acid cycle (Fig. S2A and S2B).

Consistent with a previous report [6], we found multiple succinylation sites on hydroxyacyl-CoA dehydrogenase trifunctional multienzyme complex subunit α (HADHA), a rate-limiting enzyme involved in the fatty acid oxidation pathway, were regulated by SIRT5 (Fig. S2C and S2D). In addition to HADHA, citrate synthase (CS), a rate-limiting enzyme of the tricarboxylic acid cycle, was also succinylated and regulated by SIRT5 (Fig. S2E). These results support our hypothesis that HFD may induce post-translational modifications (PTM) on key metabolic proteins and lead to dysfunction, while hepatic SIRT5 may modify such PTMs and restore the function of these proteins.

Using immunoprecipitation and LC–MS/MS shotgun proteomics, we detected and validated the interaction between SIRT5 and TANK-binding kinase 1 (TBK1) (Fig. 2S). TBK1 has recently been implicated as a potential link between hepatic inflammation and energy metabolism [7]. Although no malonylated or succinylated TBK1 was detected in liver tissue of mice fed with HFD or ND diet (Fig. S2A and S2B), we predicted that TBK1 is a potential substrate of lysine malonylation and succinylation with a toolkit we developed [8]. SIRT5 has been reported as a modulator of TBK1. SIRT5 desuccinylates TBK1 at K38, K154, and K692 and potentiates the inflammatory response [9]. On the other hand, SIRT5 desuccinylates TBK1 at K137 and suppresses the downstream inflammatory pathways [10]. The inconsistency of the role of SIRT5 in TBK1 regulation might be due to the complicated signaling pathways mediated by TBK1. The combination of succinylation at different K residues might interfere with either the protein complex formation or the kinase activity, and thus shows diverse downstream effects. We observed elevated TBK1 phosphorylation in the liver of HFD mice (Fig. 2T), indicating the chronic inflammation in MASLD models. Notably, overexpression of SIRT5 suppressed TBK1 phosphorylation (Figs. 2U and S2F) and downregulated the expression of proinflammatory cytokines (Figs. 2V and S2G ), suggesting that SIRT5 may function as a negative regulator of chronic hepatic inflammation.

Collectively, our study demonstrated a protective role of SIRT5 on the metabolic abnormality during the progression of MASLD (Fig. 2W). FFAs and proinflammatory factors upregulated the E3 ubiquitin ligase TRIM21, which in turn mediates the downregulation of SIRT5. Down-regulated SIRT5 expression led to increased lysine succinylation and malonylation in metabolism-related proteins, resulting in the dysregulation of glucose and lipid metabolism. Hepatic overexpression of SIRT5 eliminated the hyper-succinylation and hyper-malonylation, and restored the metabolic flux. Of note, TBK1, a key mediator linking metabolism and inflammation, is also a potential target of SIRT5. SIRT5 attenuated TBK1-mediated inflammation by fine-tuning the crosstalk between lysine acylation and serine phosphorylation. The results broaden the current understanding of SIRT5 as a key regulator in PTM and metabolic diseases.

## Research limitations

The mouse model of HFD and MCD diet-induced MASLD used in this study may not fully recapitulate the complexity of human MASLD. The precise regulatory effects of TBK1 activation by SIRT5 during the progression of MASLD remain to be further elucidated. Additionally, the study did not explore the long-term effects or potential side effects of SIRT5, which are critical considerations for assessing its translational and clinical applicability.

## Supplementary Material

lnag001_Supplementary_Data

## Data Availability

All data necessary to evaluate the conclusions of this study are provided in the manuscript or the Supplementary Materials. Any additional details supporting the findings of this study are available from the corresponding author upon reasonable request.
