# Supplementary material for: SIRT5 mitigates metabolic abnormality in murine models of metabolic dysfunction-associated steatotic liver disease
Source: Life Med. 2026 Jan 20;5(2):lnag001. doi: 10.1093/lifemedi/lnag001 (PMC13109097; doi:10.1093/lifemedi/lnag001)
Supplement: lnag001_Supplementary_Data [file lnag001_supplementary_data.zip › SIRT5_HFD_SI-20260115_PE.docx]

SIRT5 mitigates metabolic abnormality in murine models of metabolic dysfunction-associated steatotic liver Disease

Min Xiao^1,#^, Juncheng Zhao^1,6,#^, Zixuan Dou^2,#^, Xiangyu Chen^1,6^, Sunyuntao Xu^1^, Yu Zhang^1,6^, Hongxuan Fan^3^, Xudong Chen^4^, Ping Zhang^3^, Zhen Huang^5,^*, Boda Zhou^3,^* and Taotao Wei^1,6,^*

^1^State Key Laboratory of Biomacromolecules, Institute of Biophysics, Chinese Academy of Sciences, Beijing 100101, China

^2^Department of Rare Diseases and State Key Laboratory of Complex Severe and Rare Diseases, Peking Union Medical College Hospital, Peking Union Medical College & Chinese Academy of Medical Science, Beijing, 100730, China

^3^Department of Cardiology, Beijing Tsinghua Changgung Hospital, School of Clinical Medicine, Tsinghua University, Beijing 102218, China

^4^Ministry of Education Key Laboratory of Protein Science, Beijing Advanced Innovation Center for Structural Biology & Frontier Research Center for Biological Structure, Tsinghua-Peking Joint Center for Life Sciences, School of Life Sciences, Tsinghua University, Beijing 100084, China

^5^ Department of Hepatobiliary Surgery, National Cancer Center/National Clinical Research Center for Cancer/Cancer Hospital, Chinese Academy of Medical Sciences & Peking Union Medical College, Beijing 100021, China

^6^College of Life Sciences, University of Chinese Academy of Sciences, Beijing 100049, China

^#^These authors contributed equally to this work.

*Correspondence: weitt@ibp.ac.cn (T.W.), zhouboda@tsinghua.edu.cn (B.Z.), zhen.huang@cicams.ac.cn (Z.H.)

**Supplemental figures and figure legends**

**
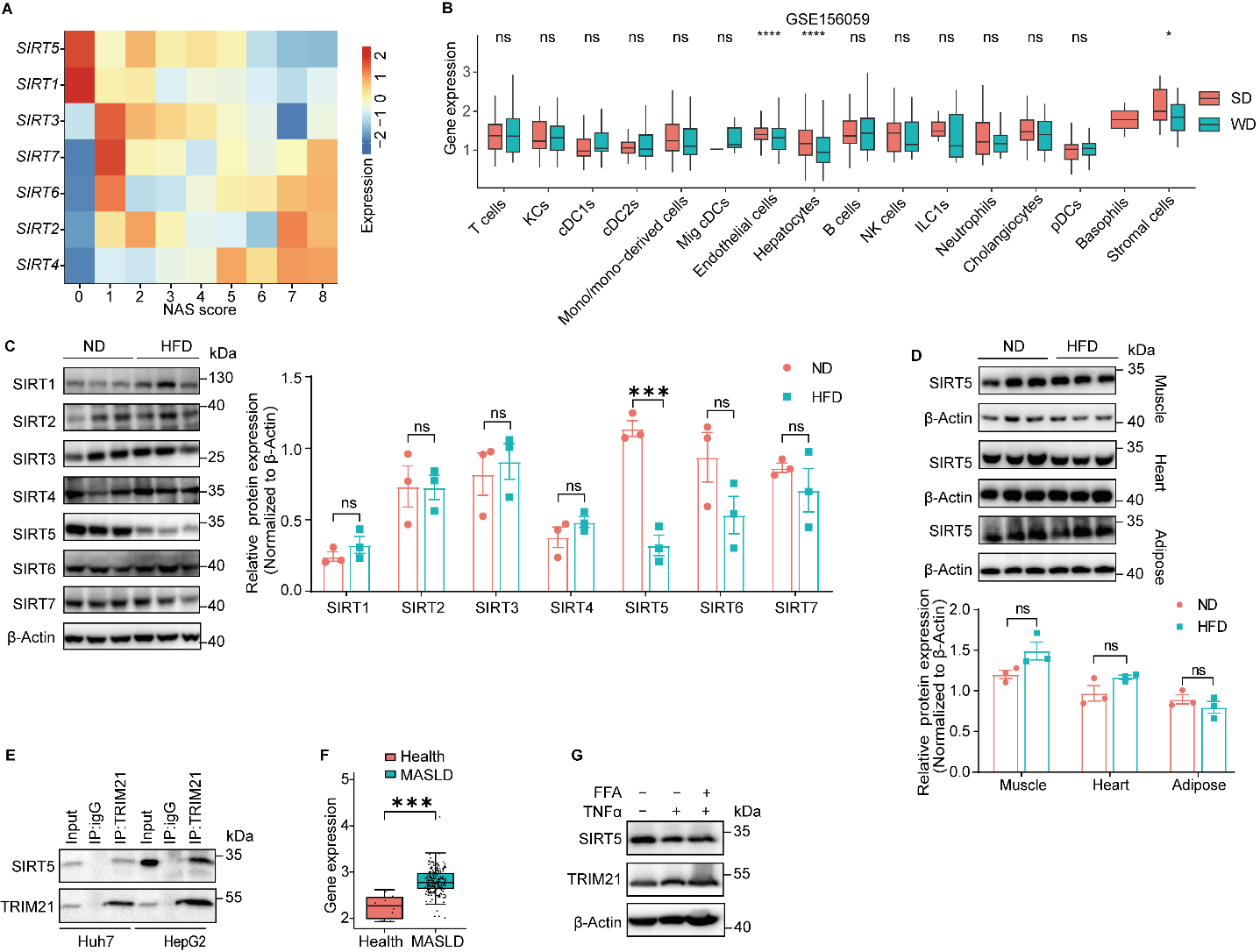
**

**Figure S1. SIRT5 is downregulated in MASLD.**

(A) Heatmap of *Sirtuin* family gene expression (*SIRT1*–*SIRT7*) in the GSE135251 MAFLD dataset. (B) *Sirt5* expression across liver cell types in WD and SD mice in the GSE156059 single‑cell RNA‑seq dataset. (C) Western blot analysis and quantification of hepatic Sirtuins in C57BL/6J mice after 14 weeks on ND or HFD (*n* = 3). ****p* < 0.001 compared to ND. (D) Western blot analysis and quantification of SIRT5 in muscle, heart and adipose of C57BL/6J mice after 14 weeks on ND or HFD (*n* = 3). (E) Lysates from Huh7 and HepG2 cells were immunoprecipitated with an anti‑TRIM21 antibody or control IgG. (F) *TRIM21* mRNA levels in liver samples from the GSE135251 MASLD cohort. ****p* < 0.001 compared to health group. (G) Western blot analysis of SIRT5 and TRIM21 expression in Huh7 cells treated with FFA mixture (200 µmol/L PA and 400 µmol/L OA) and TNF-α (50 ng/mL) for 72 h.


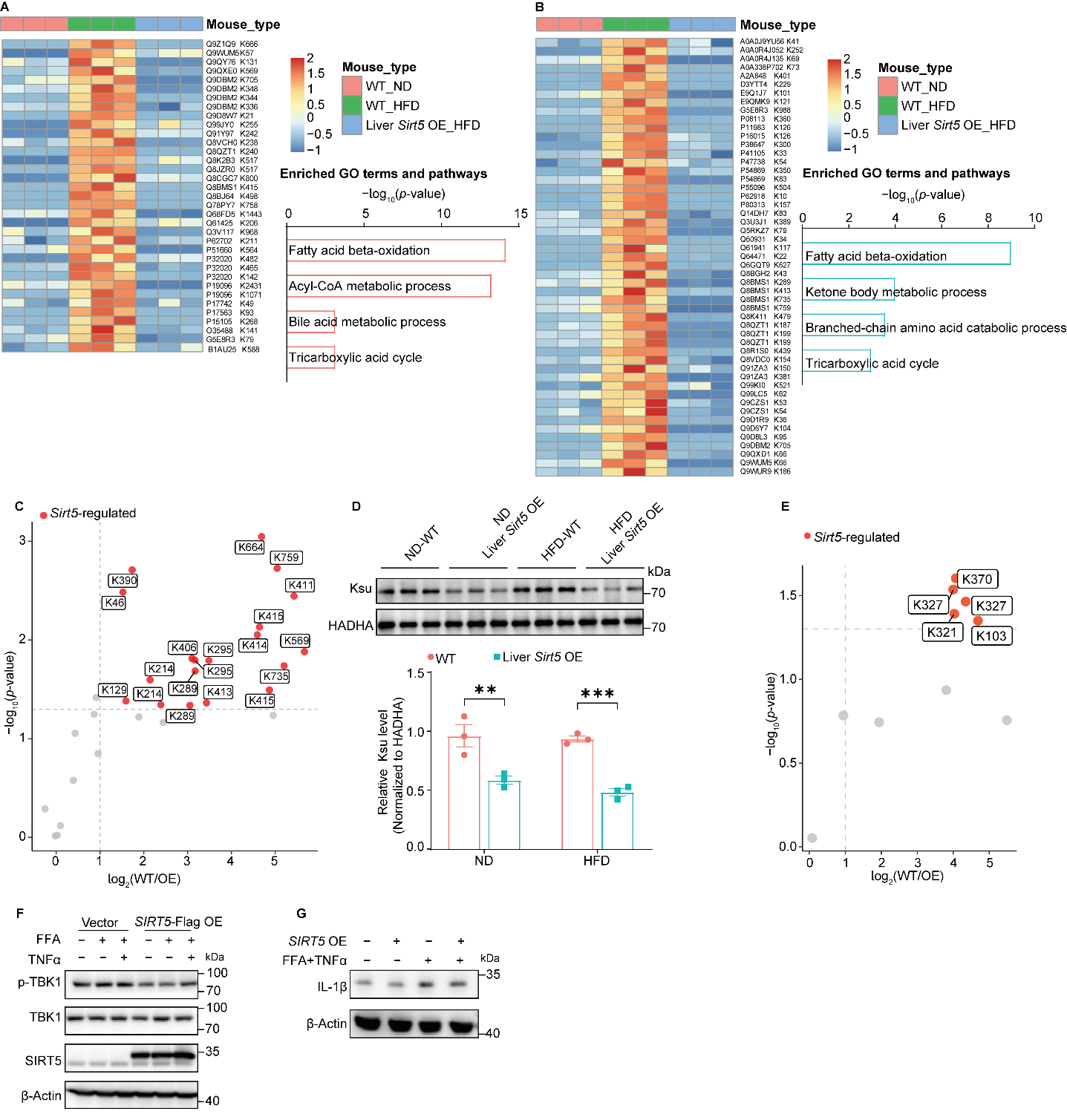


**Figure S2. Hepatic overexpression of *SIRT5* modulates the malonylome and succinylome.**

(A) Heatmap of the liver malonylome across WT mice on normal diet (WT_ND), WT mice on high‑fat diet (WT_HFD), and Liver *Sirt5* OE mice on high‑fat diet (Liver *Sirt5* OE_HFD) (*n* = 3). Colors indicate the relative abundance of lysine malonylation (Kmal) sites (blue, decreased; red, increased). Gene Ontology (GO) and pathway enrichment analysis was performed for proteins with altered Kmal. (B) Heatmap of the liver succinylome across WT mice on normal diet (WT_ND), WT mice on high‑fat diet (WT_HFD), and Liver *Sirt5* OE mice on high-fat diet (Liver *Sirt5* OE_HFD) (*n* = 3). Colors indicate the relative abundance of lysine succinylation (Ksu) sites (blue, decreased; red, increased). GO and pathway enrichment analysis was performed for proteins with altered Ksu. (C) Distribution of fold changes at lysine succinylation (Ksu) sites in the HADHA protein. Red dots denote *Sirt5*‑regulated sites, defined as a WT/OE fold change ≥ 2 with *p* < 0.05. (D) Western blot analysis of hydroxyacyl-CoA dehydrogenase trifunctional multienzyme complex subunit α (HADHA) lysine succinylation (Ksu) in liver lysates from WT and Liver *Sirt5* OE mice after 14 weeks on normal diet (ND) or high‑fat diet (HFD) (*n* = 3). ** *p* < 0.01 compared to HFD-WT group. (E) Distribution of fold changes at lysine‑succinylation (Ksu) sites in the citrate synthase (CS) protein. Red dots denote *Sirt5*‑regulated sites, defined as a WT/OE fold change ≥ 2 with *p* < 0.05. (F) Western blot analysis of phosphorylated TBK1 (p‑TBK1) in HepG2 overexpressing *SIRT5* (*SIRT5* OE) and control cell (Vector) treated with fatty acid mixture (FA; 200 µM PA and 400 µM OA) and TNF-α (50 ng/mL) for 24 h. (G) Western blot analysis of IL-1β in HepG2 overexpressing *SIRT5* (*SIRT5* OE) and control cell (Vector) treated with fatty acid mixture (FA; 200 µM PA and 400 µM OA) and TNF-α (50 ng/mL) for 24 h.

**Materials and Methods**

**Antibodies and reagents**

Antibodies against SIRT5 (15122-1-AP), HADHA (10758-1-AP), SIRT3 (10099-1-AP), SIRT4 (66543-1-Ig), SIRT6 (13572-1-AP), TRIM21 (12108-1-AP), β-tubulin (10068-1-AP) and β-actin (66009-1-Ig) were purchased from ProteinTech (Cambridge, UK). Anti-malonyllysine (PTM-901), anti-succinyllysine (PTM-419), anti-malonyllysine antibody conjugated agarose beads (PTM-902) and anti-succinyllysine antibody conjugated agarose beads (PTM-402) were purchased from PTM Biolabs (Hangzhou, China). Antibodies against SIRT1 (A11267), SIRT2 (A0273), SIRT7 (A0979), Flag-Tag (AE005), HA-Tag (AE105), IL-1β (A1112) and TBK1 (A3458) were purchased from Abclonal (Wuhan, China). Antibody against p-TBK1 (5483S) was purchased from Cell Signaling Technology (MA, USA). Anti-Flag tag magnetic beads (B26101) or anti-HA tag magnetic beads (B26201) were purchased from Selleck (TX, USA). Glucose (G7021), sodium palmitate (P9767), Oil Red O (O0625), collagenase type 4 (C5138) and sodium oleate (O7501) were purchased from Sigma-Aldrich (Missouri, USA). Insulin (P3376) was purchased from Beyotime (Shanghai, China). RIPA lysis buffer (R0020, R0030), protein loading buffer (5×) (P1040), Percoll (P8370) and Triglyceride Content Assay Kit (BC0625) were purchased from Solarbio (Beijing, China). Protease Inhibitor Cocktail (K4001), Phosphatase Inhibitor Cocktail (K1014), Deacetylase Inhibitor Cocktail (K1017) were purchased from APExBIO (TX, USA). LipidTOX Neutral Lipid Stain (H34350) and Pierce BCA Kit (23225) were purchased from Thermo Fisher (MA, USA). Total RNA extraction kit (DP419) was purchased from Tiangen Biotech (Beijing, China). PrimeScript RT Reagent Kit with gDNA Eraser (RR047A) was purchased from Takara Bio (Shiga, Japan). UltraSYBR Mixture (CW2601) was purchased from CWBIO (Beijing, China). A list of other chemicals used for LC-MS/MS can be found in our paper published previously [1].

**Animals**

C57BL/6J mice were purchased from Vital River Co., Ltd. (Beijing, China). Hepatic *Sirt5*-overexpressing mice on a C57BL/6 background (Liver *Sirt5* OE) were established by using CRISPR/Cas9 gene editing tool we described previously [2]. Males of this strain aged 6 to 20 weeks were used for the experiments in the current work. Mouse studies were approved by the ethics committee of Institute of Biophysics, Chinese Academy of Sciences (approval number: SYXK2020064), following the National Health and Medical Research Council of China Guidelines on Animal Experimentation.

**Normal diet (ND) and high-fat diet (HFD)**

Wild-type or Liver *Sirt5* OE C57 mice were fed HFD (60% fat calories, 20% protein calories and 20% carbohydrate calories) (D12492, Research Diets, NJ, USA) or ND. In most assays, the mice were fed HFD for 14 weeks. All mice were housed with 3 to 5 animals per cage, and were maintained on at 22 °C in a 12/12 h light/dark cycle in a specific pathogen-free facility and given free access to food and water.

**The glucose tolerance test (GTT) and insulin tolerance test (ITT)**

Glucose tolerance tests were performed by injecting mice intraperitoneally (i.p.) with glucose (1 g/kg body weight) after 10 h of fasting. After fasting mice for 6 h, insulin tolerance tests were performed by i.p. injection with insulin (0.5 units per kg body weight). Blood glucose levels were recorded before and at 0, 15, 30, 60, 90, and 120 min after injection and examined by an ACCU-CHEK^®^ blood glucose meter along with test strips (05942861001) from Roche Diagnostics (Basel, Switzerland).

**Serum biochemistry analysis**

Blood samples were collected from mice by retro-orbital bleeding, allowed to clot at room temperature for 30 min, and centrifuged at 3000× *g* for 15 min at 4°C to obtain serum. Biochemical parameters including triglycerides (TG), total cholesterol (TC), high-density lipoprotein cholesterol (HDL-C), low-density lipoprotein cholesterol (LDL-C), glucose, alanine aminotransferase (ALT), and aspartate aminotransferase (AST) were measured using an automatic biochemical analyzer.

**Liver histological analysis**

Mouse liver tissues were harvested rapidly, fixed, and embedded. Paraffin sections (5 μm) were cut and mounted on glass slides for hematoxylin and eosin (HE) staining. Frozen liver sections (4 μm) were stained for lipids using Oil Red O. Total lipids (mainly triglyceride, TG) in the liver were measured using commercial kits according to the manufacturer’s instructions.

**Generation of *SIRT5*-****overexpressing cell**

HEK293T cells were co-transfected with lentiviral overexpression vectors, as well as the packaging plasmids psPAX2 and pMD2.G. Supernatants containing lentiviral particles were collected after 48 h and 72 h. Target cells were infected with viral supernatants in the presence of 8  µg/mL polybrene and subsequently selected with puromycin (2 µg/mL) for 3 days to establish stable *SIRT5*-overexpressing cell lines. Overexpression efficiency was verified by qPCR and immunoblotting.

**Cell culture and fatty acids (FA) treatment**

Huh7 cells were obtained from JCRB. HepG2 cells were obtained from ATCC. Huh7 and HepG2 cells were cultured at 37℃ under an atmosphere of 5% CO₂ in Dulbecco’s Modified Eagle’s Medium (DMEM) containing 10% fetal bovine serum (FBS) and 1% penicillin/streptomycin (P/S). The culture medium was replaced every 48 h. When reaching approximately 80%–90% confluence, cells were digested and cultured overnight in appropriate plates.

Fatty acid stock solutions (palmitate or oleic acids) were complexed with fatty acid-free BSA in cell culture medium. Briefly, sodium palmitate and sodium oleate were each dissolved in 0.1 mol/L NaOH solution at 75℃ for 30 min. 20% fatty acid-free BSA was dissolved in ddH_2_O and incubated at 55℃ for 30 min. The sodium palmitate and the sodium oleate were mixed each with BSA to make 10 mmol/L palmitate-BSA and oleate-BSA stock solution, and then filtered and stored at 4℃. Cells were incubated with FA-BSA complexes for the indicated time, while BSA alone was used as a vehicle control.

**RNA extraction and real-time PCR**

Liver tissue or cell RNA was extracted using RNA simple Total RNA Kit according to the manufacturer’s instructions. cDNA was synthesized using a PrimeScriptTM RT Reagent kit. Real-time PCR was performed using the UltraSYBR Mixture on the QuantStudioTM 6 Flex Real-Time PCR System (Applied Biosystems, 4484642). Relative changes in mRNA expression were calculated using the comparative cycle threshold method (2^−ΔΔCt^). The specific primer sequences used for real-time PCR were listed in Table S1.

**Isolation and culture of primary hepatocytes**

Mice were anesthetized and the abdominal cavity was dissected. The liver was perfused with KRG buffer (120 mmol/L NaCl, 20 mmol/L NaHCO₃, 20 mmol/L glucose, 5 mmol/L HEPES (pH 7.4), 5 mmol/L KCl, 1 mmol/L MgSO₄, 1 mmol/L KH₂PO₄) with 0.5 mmol/L EGTA through the visceral vena cava. When the blood was drained, the solution was switched to KRG buffer with 25 mg/mL collagenase type 4. The liver was cut to release the hepatocytes. The hepatocytes were washed with ice-cold DMEM and the suspension was passed through a 70 μm cell strainer and centrifuged at 800× *g* for 3 min. The 25% Percoll/hepatocytes solution was prepared and centrifuged at 1800× *g* for 5 min. After carefully removing the supernatant, the cell pellet was washed to remove the Percoll. The wash step was repeated until the supernatant appeared clear after centrifugation. The isolated mouse hepatocytes were then plated in type I collagen-coated 6-well plates.

**Seahorse assay**

Oxygen consumption rates (OCRs) detection of cells were detected with the Seahorse XF24 analyzer (Agilent, CA, USA) according to the manufacturer’s protocol. A total of 20,000 cells in 100 μL cell culture medium were seeded in each well of an XF 24-well cell culture microplate and cultured overnight at 37 °C in 5% CO₂. As a negative control, four wells were kept devoid of cells and given only Seahorse media, which comprises basal XF media, 5.5 mmol/L glucose, 1 mmol/L sodium pyruvate and 4 mmol/L glutamine (additionally, the pH was adjusted to 7.4). Twelve hours prior to running a plate, the Seahorse sensor cartridge was incubated with Seahorse Calibrant solution according to the manufacturer’s protocol, in a 37°C, CO₂-free incubator. On the day of an assay, cells were washed and incubated with Seahorse media. The sensor cartridge was fitted onto the cell culture plate, which was then placed into a 37°C, CO₂-free incubator for 1 h. The assay was run on the Seahorse XF24 Analyzer. The three pharmaceutical modulators of mitochondrial OXPHOS were used for OCR measurement: oligomycin (1 μmol/L), FCCP (1 μmol/L), rotenone and Antimycin A (1 μmol/L). For the FAO measurement in cells, cells were seeded in XF24 Cell Culture Microplates and cultured overnight in cell culture medium. The following day, growth medium was replaced with substrate-limited medium and the cells incubated for an additional 24 h. Forty-five min prior to the assay, the cells were washed one to two times with FAO assay medium. A volume of 375 μL per well of FAO assay medium was added to the cells, which were then incubated in a non-CO₂ incubator at 37 °C for 30 min. Fifteen min prior to the start of the assay, etomoxir was diluted to 400 μmol/L in FAO assay medium. Palmitate-BSA (200 μmol/L) was added before running the assay. During the assay, the three pharmaceutical modulators of mitochondrial OXPHOS were used for OCR, FAO-dependent OCR was defined as the (OCR untreated with etomoxir)–(OCR treated with etomoxir).

**Western blotting analysis**

Cells and homogenized tissues were lysed with RIPA lysis buffer containing 1× protease inhibitor and 1× phosphatase inhibitor for 20 min on ice, then centrifuged at 12,000× *g* for 15 min at 4°C, and the supernatant was collected. The protein concentration was quantified with Pierce BCA Kit. Protein lysates were separated by SDS–PAGE and transferred onto PVDF membranes. Membranes were blocked and incubated with primary antibodies overnight at 4℃, followed by horseradish peroxidase-conjugated secondary antibodies for 1  h at room temperature. Signals were detected using the ChemiDoc XRS system (Bio-Rad, CA, USA), and band intensities were quantified with Image Lab software.

**Co-immunoprecipitation and immunoprecipitation-MS**

For exogenous Co-IP analysis, cells were transiently co-transfected with corresponding plasmids. After 48 h, cells were lysed in ice-cold lysis buffer (50 mmol/L Tris–HCl (pH 7.4), 150 mmol/L NaCl, 1% NP-40, protease inhibitor cocktail). Cell lysates were cleared by centrifugation and incubated with magnetic beads conjugated with anti-Flag antibody or anti-HA antibody overnight at 4°C. After extensive washing, the beads were resuspended with 1× SDS loading buffer and denatured at 95°C for 10 min. After the magnetic beads were removed, Co-IP samples were prepared for subsequent Western blotting.

For endogenous Co-IP analysis, cells or liver tissues were homogenized in lysis buffer as described above. Lysates were incubated with target-specific primary antibodies overnight at 4°C, together with normal IgG as a negative control. Antibody–protein complexes were captured by protein A/G magnetic beads, washed thoroughly, and subjected to SDS–PAGE followed by immunoblotting with appropriate detection antibodies.

For IP-MS, cell lysates were subjected to immunoprecipitation with anti-Flag magnetic beads as described above. Bound proteins were eluted by boiling in SDS sample buffer and separated by SDS–PAGE. Gels were stained with Coomassie Brilliant Blue, and the lanes corresponding to immunoprecipitated protein complexes were excised into gel slices. After decolorization, DTT reduction and iodoacetamide alkylation, trypsin was added for overnight, and then the peptide was extracted with acetonitrile at multiple steps with different concentrations. The resulting peptides were analyzed by liquid chromatography–tandem mass spectrometry (LC–MS/MS) on a Q Exactive Plus mass spectrometer (Thermo Fisher, MA, USA). Database searching and protein identification were performed in the Uniprot_proteome_Human_2022 database using the Thermo Proteome Discoverer software (version 2.2.0.388), and protein identification was performed with a false discovery rate (FDR) < 1%.

**Immunofluorescence microscopy**

Lipid droplets were visualized using the LipidTOX Neutral Lipid Stain according to the manufacturer’s instructions. Briefly, cells were cultured on glass coverslips and treated as indicated, then washed twice with PBS and fixed in 4% paraformaldehyde for 15  min at room temperature. After rinsing with PBS, cells were incubated with LipidTOX dye (1:500 dilution in PBS) for 30 min at room temperature in the dark. Nuclei were counterstained with DAPI for 5  min, and samples were mounted with antifade mounting medium. The samples were kept in the dark and the fluorescence signals were detected by confocal laser scanning microscope FV3000 series (Olympus, Tokyo, Japan).

**Transmission electron microscopy**

Mice were euthanized, and then the liver was exposed with sharp surgical scissors. Liver tissue (1 mm × 1 mm × 5 mm) was cut, transferred into a 2-mL polypropylene microcentrifuge tube containing prefixation solutions (2.5% glutaraldehyde, 0.8% paraformaldehydein, 0.1 mol/L sodium phosphate buffer, pH 7.4), and fixed for 1h at 25℃. After overnight at 4℃, samples were treated with 0.1 mol/L imidazole, post-fixed in 2% osmium tetroxide, and stained with 1% uranyl acetate at 4℃ overnight. Tissues were dehydrated through a graded acetone series, embedded in epoxy resin (60℃, 24 h). Samples were sectioned using Leica EM UC7 (about 60 nm thick, Wetzlar, Germany) and placed on copper grids. Images were recorded on the transmission electron microscope Spirit 120 kV (ultra-thin slices).

**Mass spectrometry**

Mice liver tissues were homogenized in RIPA buffer containing Protease Inhibitor Cocktail, then centrifuged at 12,000× *g* at 4℃ for 15 min. Proteins in the supernatant were precipitated with acetone and resolved in 8 mol/L urea containing 50 mmol/L Tris–HCl, pH 8.0. Tryptic digestion and affinity enrichment of acylated peptides were performed as previously described [1]. The malonyllysine and succinyllysine peptides were analyzed by reverse phase LC–MS/MS, and then SEQUEST HT search engine with Thermo Proteome Discoverer (version 2.2.0.388) in Uniprot proteome mouse database (update-180920) for protein identification. The normalization to the protein median of each sample was used to correct experimental bias and the normalization mode was selected as total peptide amount. Protein ratios were calculated as the median of all possible pairwise ratios. Missing values were imputed using random forests model. GO functional enrichment analysis (*p* value <0.05) was carried out by using the ClusterProfiler package in R software. Normalized values were used to generate heatmaps using the pheatmap R-package in R (version 3.61).

**Public datasets analysis**

Gene Expression Omnibus (GEO) datasets (GSE37031, GSE63067, GSE135251) comprising clinical liver biopsy samples from patients with steatohepatitis and non‑steatohepatitis controls were analyzed to assess differential expression of the Sirtuin family (*SIRT1–SIRT7*). The gene expression data were processed by quantile normalization and log_2_-transformation, and between-group differences were assessed using the Wilcoxon rank-sum test. Sirtuin family (*SIRT1–SIRT7*) gene expression data were retrieved from GEO dataset GSE135251. Samples were stratified by Steatohepatitis Activity Score (NAS) into distinct groups. Expression values were normalized and log_2_-transformed. A clustered heatmap was generated to visualize and compare group-wise expression patterns, identifying differential expression trends between NAS groups.

**Statistical analysis**

Descriptive data were presented as the mean ± standard error of the mean (SEM) for continuous variables. Data were analyzed with an independent *t*-test or one-way analysis of variance for continuous variables. Significance was assumed at a two-sided *p* value < 0.05. Statistical analysis was performed using GraphPad Prism (version 8.3.1).

**Data availability**

All data necessary to evaluate the conclusions of this study are provided in the manuscript or the Supplementary Materials. Any additional details supporting the findings of this study are available from the corresponding author upon reasonable request.

**References**

1. Du Y, Cai T, Li T, et al. Lysine malonylation is elevated in type 2 diabetic mouse models and enriched in metabolic associated proteins. *Mol Cell Proteomics* 2015; 14(1): 227-236.
2. Zhou B, Xiao M, Hu H, et al. Cardioprotective role of SIRT5 in response to acute ischemia through a novel liver-cardiac crosstalk mechanism. *Front Cell Dev Biol* 2021; 9: 687559.
